# The role of cardiovascular autonomic failure in the differential diagnosis of α-synucleinopathies

**DOI:** 10.1007/s10072-021-05746-6

**Published:** 2021-11-24

**Authors:** Fabian Leys, Gregor K. Wenning, Alessandra Fanciulli

**Affiliations:** Division of Neurobiology, Department of Neurology, Medical University of Innsbruck, Anichstrasse 35, Innsbruck, 6020 Austria

**Keywords:** Autonomic nervous system, Orthostatic hypotension, Cardiovascular autonomic failure, Differential diagnosis, Synucleinopathies

## Abstract

The α-synucleinopathies comprise a group of adult-onset neurodegenerative disorders including Parkinson’s disease (PD), multiple system atrophy (MSA), dementia with Lewy bodies (DLB,) and — as a restricted non-motor form — pure autonomic failure (PAF). Neuropathologically, the α-synucleinopathies are characterized by aggregates of misfolded α-synuclein in the central and peripheral nervous system. Cardiovascular autonomic failure is a common non-motor symptom in people with PD, a key diagnostic criterion in MSA, a supportive feature for the diagnosis of DLB and disease-defining in PAF. The site of autonomic nervous system lesion differs between the α-synucleinopathies, with a predominantly central lesion pattern in MSA versus a peripheral one in PD, DLB, and PAF. In clinical practice, overlapping autonomic features often challenge the differential diagnosis among the α-synucleinopathies, but also distinguish them from related disorders, such as the tauopathies or other neurodegenerative ataxias. In this review, we discuss the differential diagnostic yield of cardiovascular autonomic failure in individuals presenting with isolated autonomic failure, parkinsonism, cognitive impairment, or cerebellar ataxia.

## Introduction

The α-synucleinopathies are a group of adult-onset, progressive neurodegenerative disorders characterized by intracellular aggregation of misfolded α-synuclein in the peripheral and central nervous system (CNS), including Parkinson’s disease (PD), multiple system atrophy (MSA), and dementia with Lewy bodies (DLB) [1, 2]. Oligodendroglial cytoplasmic α-synuclein inclusions are characteristic of MSA, which is hence considered an oligodendroglial α-synucleinopathy [3]. Depending on the predominance of either cerebellar signs or parkinsonism, a cerebellar (MSA-C) and a Parkinsonian variant (MSA-P) are distinguished [4]. Neuronal α-synuclein inclusions, the so-called Lewy bodies and neurites, represent the pathological hallmark of PD and DLB, which are therefore referred to as the Lewy body disorders [5]. Lewy body pathology with deposition of α-synuclein in sympathetic ganglia and peripheral autonomic fibers was also found in individuals with pure autonomic failure (PAF) [6, 7], tying it to the group of α-synucleinopathies as a restricted non-motor form [2].

**Fig. 1:**
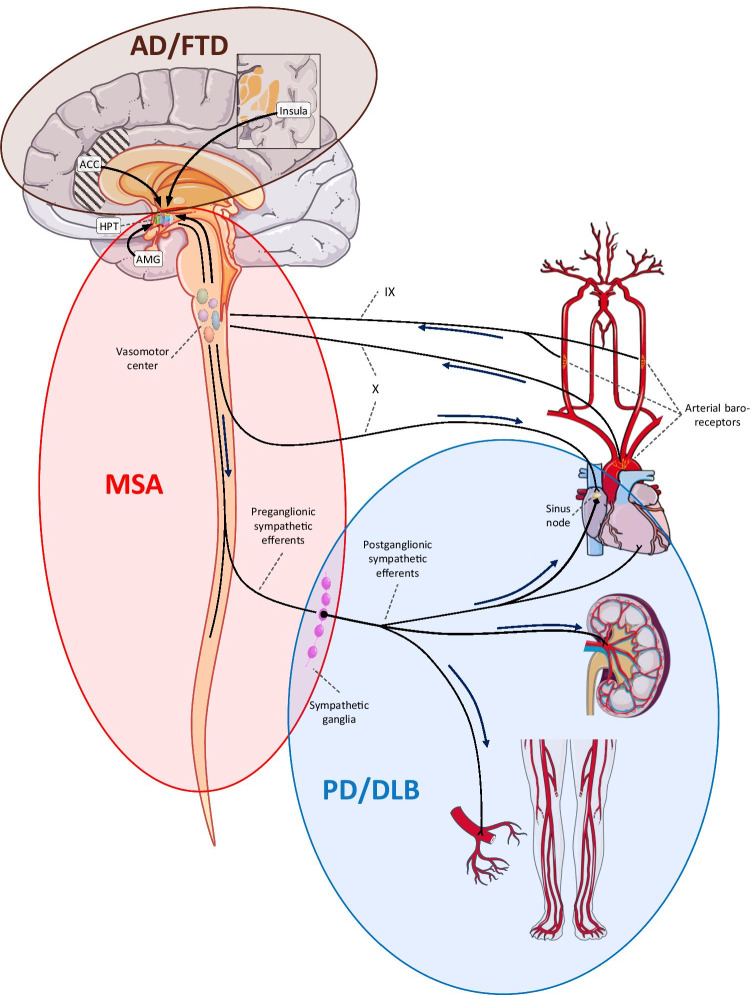
The cardiovascular autonomic system with disease-specific sites of lesion. Afferent, central, and efferent components of the cardiovascular autonomic network with possible sites of lesion causing cardiovascular autonomic failure. Deposition of hyperphosphylated τ-protein and β-amyloid plaques in central autonomic relays, such as the ACC, AMG, and insula presumably cause cardiovascular autonomic impairment in AD and FTD; predominantly central/pre-ganglionic deposition of oligodendroglial cytoplasmic α-synuclein inclusions occurs in MSA, while PD and DLB are characterized by predominantly peripheral/post-ganglionic deposition of neuronal α-synuclein inclusions. Central structures, particularly the dorsal motor nucleus of vagus, are also affected in PD/DLB, whereas in MSA the neurodegenerative process may propagate from the pre- to the post-ganglionic branch during the disease course. *ACC* anterior cingulate cortex; *AD* Alzheimer’s disease; *AMG* amygdala; *DLB* dementia with Lewy bodies; *FTD* frontotemporal dementia; *HPT* hypothalamus; *IX* glossopharyngeal nerve; *MSA* multiple system atrophy; *PD* Parkinson’s disease; *X* vagal nerve. Created with Microsoft Office PowerPoint 2016, in part using adapted Servier Medical Art images. (https://smart.servier.com/)

Orthostatic hypotension (OH) is defined by consensus as a sustained fall in systolic/diastolic blood pressure (BP) of ≥ 20/10 mmHg within three minutes upon postural change [8]. Orthostatic BP falls causing recurrent spells of dizziness, light-headedness, pre-syncope, or syncope episodes may occur for various reasons [9]. In disorders such as the α-synucleinopathies, however, OH is mostly due to neurodegenerative changes affecting the cardiovascular autonomic nervous system (ANS), i.e. it is of neurogenic origin [10]. The site of ANS lesion is mainly central, or pre-ganglionic, in MSA [3] and peripheral, or post-ganglionic, in PD and DLB (Fig. 1) [11, 12].

Cardiovascular autonomic function tests (CAFT) represent the gold standard for diagnosing neurogenic OH, which is characterized by an insufficient compensatory heart rate (HR) increase despite severe orthostatic BP falls and missing BP counter regulation under baroreflex stimulation, like during the Valsalva manoeuver [13, 14]. Neurogenic OH affects every third person with PD [15, 16], it is a key diagnostic criterion for MSA [4, 17, 18], supports the diagnosis of DLB [19–21], and defines the disease in PAF [22]. High BP levels while supine and asleep, i.e. supine and nocturnal hypertension [23], post-prandial hypotension [24] and exercise-induced hypotension [25] may accompany neurogenic OH in about 50% of individuals, with major prognostic and therapeutic implications [10].

Distinguishing one α-synucleinopathy from the other, but also separating them from related disorders, such as the tauopathies or other neurodegenerative ataxias, is often challenging, especially at disease onset due to overlapping presentation [26, 27]. Misdiagnosis may result in inadequate treatment, erroneous or missed inclusion in clinical trials. Given the narrow window of opportunity in which candidate neuroprotective therapies might help modifying the relentless disease progression of the α-synucleinopathies, a timely and accurate diagnosis is of utmost importance for an appropriate counselling and improved health care.

This review focuses on the role of cardiovascular autonomic failure in the differential diagnostic work-up of individuals presenting with neurological features that potentially indicate an incipient α-synucleinopathy, i.e. isolated autonomic failure, parkinsonism, cognitive impairment, or cerebellar ataxia.

## Methods

References were identified by searching the PubMed database up to April 2021, combining keywords for disease entities, disorders, or non-ANS-related clinical features with ANS-related and other keywords as follows: [“Alzheimer dementia” OR “Alzheimer disease” OR “ataxia” OR “CANVAS” OR “cerebellar ataxia” OR “cognitive impairment” OR “corticobasal degeneration” OR “corticobasal syndrome” OR “dementia with Lewy bodies” OR “dementia” OR “fragile X tremor ataxia syndrome” OR “Friedreich ataxia” OR “frontotemporal dementia” OR “Lewy body disorders” OR “multiple system atrophy” OR “neurodegenerative dementia” OR “parkinsonism” OR “Parkinson’s disease” OR “progressive supranuclear palsy” OR “pure autonomic failure” OR “spinocerebellar ataxia” OR “sporadic adult onset ataxia” OR “sporadic late onset ataxia” OR “synucleinopathies” OR “tauopathies”] AND [“autonomic” OR “autonomic dysfunction” OR “autonomic failure” OR “autonomic function” OR “cardiovascular autonomic dysfunction” OR “cardiovascular autonomic failure” OR “differential diagnosis” OR “exercise induced hypotension” OR “heart rate variability” OR “isolated autonomic failure” OR “natural history” OR “neurogenic orthostatic hypotension” OR “nocturnal hypertension” OR “orthostatic hypotension” OR “postprandial hypotension” OR “supine hypertension” OR “syncope”].

Meta-analyses, systematic and narrative reviews, cross-sectional, prospective and retrospectives original studies, as well as case reports, were included. Additional data were collected by hand-searching references from selected articles. We also included historically noteworthy and conceptually related articles. We only considered articles published in English language.

## Isolated autonomic failure

PAF was defined by consensus in 1996 as a sporadic disorder characterized by idiopathic neurogenic OH with evidence of more widespread autonomic failure, but no other neurological features [22]. At this time, it was already known that low supine plasma norepinephrine (NE) levels, indicating a widespread post-ganglionic sympathetic denervation, are characteristic of PAF [6, 7], as well as that some individuals will eventually develop a manifest CNS involvement during the disease course [22]. For this reason, a history of at least five years of isolated autonomic failure without development of other neurological features was proposed as additional criterion to increase the diagnostic accuracy for PAF [28].

In recent years, one prospective North American multi-center study as well as three retrospective single-center studies from two independent research consortia investigated the natural history of PAF and reported a phenoconversion to another α-synucleinopathy in approximately one-third of individuals [2, 29–31]. The phenoconversion rate to MSA was 7–28% and to Lewy body disorders 4–26%, depending on the duration of the clinical follow-up (see Table 1) [2, 29–31].

**Table 1:** Natural history studies of PAF

	Kaufmann et al. [2]	Singer et al. [29]		Coon et al. [30]	Giannini et al. [31]
Design/cohort	Prospective multi-center (NYUMC, NIH, VU, Mayo Clinic, Beth Israel; US)	Retrospective single-center (Mayo Clinic, US)			Retrospective single-center (Bologna, Italy)
Published	2017	2017		2020	2018
Follow-up, years	4	any	≥ 3	≥ 5	≥ 5
Sample size, n	74	318	78	275	50
Conversion rate, n (%)	25 (34)	37 (12)^*^	37 (47)^*^	67 (24)	16 (32)^**^
Conversion to Lewy body disorders, n (%)	19 (26)^+^	11 (4)	11 (14)	33 (12)	3 (6)^++^
Conversion to MSA, n (%)	6 (8)	22 (7)	22 (28)	34 (12)	10 (20)

*DLB*, dementia with Lewy bodies; *MSA*, multiple system atrophy; *NIH*, National Institute of Health Bethesda; *NYUMC*, New York University Medical Center; *PD*, Parkinson’s disease; *US*, United States; *VU*, Vanderbilt University

^*^Including *n* = 4 individuals with development of clear motor involvement, but indeterminate diagnosis

^**^Including *n* = 3 individuals with undefined parkinsonism

^+^Including *n* = 13 (18) DLB and *n* = 6 (8) PD phenoconverters

^++^Including *n* = 2 (4) DLB and *n* = 1 (2) PD phenoconverters

Classification of phenoconversion to PD or DLB not specified in [29, 30]

Individuals who maintained the PAF phenotype (“stable PAF”) or rather phenoconverted to another α-synucleinopathy with overt CNS involvement (“CNS α-synucleinopathy”) were found to exhibit distinct baseline features, which ultimately foreshadowed the disease course [2, 29–31]. The current understanding is that features suggestive of CNS involvement, such as dream enacting behavior and subtle motor signs, as well as earlier or more severe manifestation of additional autonomic disturbances, indicate a future phenoconversion to a CNS α-synucleinopathy [2, 29–31]. Together with indices referring to either a predominant central or peripheral pattern of ANS lesion, it may be possible to predict whether an individual will eventually phenoconvert to MSA or a Lewy body disorder already at prodromal disease stages (see Table 2) [2, 29–31].

**Table 2:** Features associated with phenoconversion from PAF to MSA versus Lewy body disorders, or both

	Multiple system atrophy	Lewy body disorders
Hemodynamic indices	Earlier OH onset (mainly 50 s) [2, 30] Preserved HR increase to tilt, i.e.: - after 1 min [30]; - > 10 bpm after 3 min [2]	Later OH onset (mid to end 60 s) [2, 29, 30]
Imaging and wet biomarkers	Supine NE levels > 100 pg/ml [29, 30] or > 110 pg/ml [2] Higher orthostatic NE levels [29] Normal cardiac ^123^I-MIBG uptake^*^ [31, 32] Lower maximum-ThT-fluorescence at PMCA assay and higher Nfl levels in CSF [33] Putaminal/infratentorial abnormalities and increased putaminal/MCP diffusivity at MRI^*^ [34]	
	Orthostatic NE rise > 65 pg/ml [29, 30] Presynaptic nigrostriatal denervation at DAT-SPECT^*^ [34, 35] Loss of dorsolateral nigral hyperintensity at MRI^*^ [35]	
Other autonomic or CNS-related signs and features	Preserved olfactory function^**^ [2] Urinary dysfunction [2, 29, 30] and constipation [2] of moderate to severe degree Preganglionic sweat loss pattern [29, 30] Stridor [31]	Impaired olfactory function^**^ [2]
	Constipation [2, 30] (contrasting evidence with [31]) Earlier onset of urinary dysfunction and intermittent catheterization^***^ [31] Dream enactment behavior, i.e. probable RBD [2, 30] and earlier onset of VPSG-confirmed RBD^***^ [31] Subtle motor signs [2, 29, 30], frequent falls^***^ [31]	

*CNS*, central nervous system; *HR* heart rate; *MCP*, middle cerebellar peduncle; *MSA*, multiple system atrophy; *NE*, norepinephrine; *Nfl*, neurofilament light chain; *OH*, orthostatic hypotension; *PAF*, pure autonomic failure; *PMCA*, protein misfolding cyclic amplification; *RBD*, REM sleep behavior disorder; *ThT*, thioflavin T; *VPSG*, video-polysomnography

^*^Not systematically studied

^**^In combination with probable RBD

^***^Individuals later phenoconverting to MSA or Lewy body disorders analyzed together in [31]

In abovementioned studies, both individuals with stable PAF and future phenoconverters to Lewy body disorders had a later disease onset and showed low baseline supine plasma NE levels, as well as an insufficient orthostatic HR increase, indicating a peripheral pattern of ANS lesion [2, 29–31]. However, Lewy body phenoconverters differed from individuals with stable PAF for a preserved orthostatic NE rise (indicating partially preserved post-ganglionic sympathetic fibers), presence of subtle motor parkinsonian signs, hyposmia, as well as constipation and an earlier occurrence of urinary dysfunction (see Table 2) [2, 29–31]. On the other hand, earlier onset of neurogenic OH, preserved orthostatic HR increase, higher supine and orthostatic NE levels, a pre-ganglionic sweat loss pattern (all consistent with a central ANS lesion pattern), as well as the presence of subtle parkinsonian or cerebellar signs, stridor, more severe urinary dysfunction and constipation, but preserved olfaction, predicted future phenoconversion to MSA [2, 29–31].

Supportive imaging data are scarce, with results from cardiac ^123^I-MIBG-SPECT only available in one study [31]. Reduced ^123^I-MIBG-uptake, indicating sympathetic cardiac denervation, was observed only in individuals with stable PAF or future Lewy body phenoconverters, while MSA phenoconverters showed normal cardiac ^123^I-MIBG-uptake, indicating a pre-ganglionic site of ANS lesion [31]. This was also the case in three individuals presenting with isolated neurogenic OH, who had normal cardiac ^123^I-MIBG findings and later phenoconverted to MSA, which was neuropathologically confirmed in two [32]. The utility of DAT-SPECT and conventional MRI as predictors of phenoconversion is yet unknown [34]. Presynaptic nigrostriatal denervation as indicated by pathological DAT-SPECT findings and loss of dorsolateral nigral hyperintensity at MRI may be predictors for both MSA and Lewy body phenoconversion [35], whereas putaminal and infratentorial abnormalities, as well as increased putaminal and middle cerebellar peduncle diffusivity on MRI, may prove to be predictors of MSA phenoconversion only [34].

A very recent study prospectively followed a well-characterized cohort of PAF individuals to determine the role of α-synuclein oligomers and neurofilament light chain (Nfl) in cerebrospinal fluid as markers of phenoconversion [33]. As previously shown for the distinction of MSA from Lewy body disorders [36], higher Nfl measurements and lower maximum-thioflavin-fluorescence in the protein misfolding cyclic amplification (PMCA) assay of baseline cerebrospinal fluid accurately distinguished future phenoconversion to MSA versus Lewy body disorders or stable PAF [33].

The role of other features of cardiovascular autonomic failure, such as supine hypertension, post-prandial- or exercise-induced hypotension, in predicting future phenoconversion to a central α-synucleinopathy in people presenting with isolated autonomic failure has not been extensively studied yet, but it is probably limited.

### Parkinsonism

Parkinsonism is characterized by the presence of bradykinesia and at least one additional extrapyramidal feature out of rigidity, rest tremor or postural instability [16]. Beyond PD, which accounts for the majority of cases [37], atypical parkinsonian disorders including DLB, MSA, progressive supranuclear palsy (PSP), and corticobasal syndrome (CBS) have to be considered in the diagnostic work-up of individuals presenting with parkinsonism [38].

Both PSP and CBS belong to the tauopathies, a group of heterogeneous neurodegenerative disorders characterized by abnormal intracerebral aggregation of τ-protein in neurons and glia [39, 40]. While features of otherwise unexplained autonomic dysfunction qualify as exclusion criteria for the diagnosis of CBS [40], autonomic disturbances in PSP mainly involve the urogenital domain and are generally less common and severe compared to the α-synucleinopathies [34, 39, 41, 42]. Although incipient signs of cardiovascular dysfunction, including loss of the physiological nocturnal BP fall and post-prandial hypotension, have been reported in PSP, severe orthostatic BP falls of ≥ 30/15 mmHg do not usually occur, especially at early stages [42–46]. More recent reviews of large neuropathologically-proven PSP series found OH to occur only in single cases (in which other frequent causes of both neurogenic and non-neurogenic OH were not excluded systematically), confirming that the cardiovascular sympathetic system is largely spared in PSP [41, 47]. Beyond other clinical and instrumental features, the presence of overt cardiovascular autonomic failure therefore yields a high negative predictive value for a diagnosis of both PSP and CBS and helps distinguishing parkinsonian syndromes due to an underlying tauopathy from those due to α-synucleinopathies [34, 39–41].

On the other hand, the presence of autonomic failure may often make it difficult to differentiate one α-synucleinopathy from the other, especially at early stages, where both people with Lewy body disorders and MSA may show good l-Dopa responsiveness [16, 27, 48]. Recent neuropathological series of individuals diagnosed in-life with MSA following the Gilman 2008 criteria [4] reported a diagnostic accuracy of 62 to 79%, with Lewy body disorders accounting for the most common misdiagnosis [41, 49]. A review of medical records identified severe, widespread autonomic failure as the main reason for Lewy body disorder individuals to receive an ante-mortem diagnosis of MSA [49]. Due to the proposed different site in ANS lesion [3, 11], hemodynamic indices of CAFT were often studied for their diagnostic yield in discriminating MSA from Lewy body disorders, particularly PD [20]. Significant differences were frequently reported, but inconsistencies and substantial overlap hinder a reliable differentiation between PD and MSA based on CAFT indices alone [14, 27, 34, 50, 51]. Cardiovascular autonomic failure is generally more frequent and severe in MSA than in Lewy body disorders [15, 17, 18, 20, 46]. However, the presence of neurogenic OH remains by itself of limited diagnostic value in single cases, while its early development, in conjunction with additional urological autonomic features and rapid progression of motor impairment yields a higher accuracy for separating MSA from PD [52, 53]. By contrast, at more advanced disease stages, every third patient with PD may develop neurogenic OH and neither its presence, nor its severity remain specific of MSA [15, 52, 53].

Consistent with the predominantly peripheral ANS involvement in the Lewy body disorders [11], cardiac sympathetic denervation at ^123^I-MIBG-SPECT or ^18^F-Dopamine-PET has been shown in PD and DLB individuals with and without neurogenic OH [54–57]. In absence of cardiomyopathy or other causes of peripheral autonomic neuropathy, the presence of cardiac sympathetic denervation is therefore considered a supportive imaging criterion for both a PD and DLB diagnosis [19, 55–59]. By contrast, most individuals with MSA have an intact cardiac innervation [55–57]. Due to a possible prion-like propagation of α-synuclein pathology from the pre- to the post-ganglionic sympathetic branch [26], however, cardiac sympathetic denervation may be found in people with MSA as well, especially at advanced stages, limiting the implementation of cardiac sympathetic imaging in its diagnostic work-up [4, 55, 58].

Measurement of plasma NE levels may also be helpful to differentiate between MSA and Lewy body disorders [14]. Supine plasma NE levels in MSA are comparable to those of people with non-neurogenic OH, whereas significantly lower levels were demonstrated in individuals with neurogenic OH due to Lewy body disorders [14]. To date, however, clear cut-off values with optimized sensitivity and specificity are still missing.

Supine and nocturnal hypertension are more common and severe in individuals with neurogenic OH due to MSA than PD [23, 50, 60], and presumably DLB, but same as for post-prandial- and exercise-induced hypotension, yield a limited value for differentiating one α-synucleinopathy from the other.

### Cognitive impairment

Cognitive impairment is defined by any deterioration in cognitive performance from the individual previous level that is significant enough to interfere with the independent daily functioning [61]. Alzheimer’s disease (AD) represents the most common cause of cognitive impairment [61]. Frontotemporal dementia (FTD) and Lewy body disorders (i.e. DLB and PD dementia) respectively account for the second leading cause of neurodegenerative cognitive decline under and above the age of 65 [61, 62].

The FTD spectrum includes various clinical subtypes [62], including its behavioral variant (bvFTD). Signs of both sympathetic and cardio-vagal dysfunction have been reported in individuals with the bvFTD and AD [63–66], possibly due to affection of telencephalic structures involved in the central autonomic network (mainly the anterior cingulate- and insular cortex; see Fig. 1) [67, 68]. Compared to healthy elderly controls, a higher prevalence of post-prandial hypotension, but not of OH, was also shown in AD [69]. Supine BP readings of individuals with AD provided contrasting results as to the presence of supine hypertension, which was not found in individuals with bvFTD [64, 66]. To the best of our knowledge, there are no systematic studies on cardiovascular autonomic function in people with other FTD variants, such as agrammatic- or semantic primary progressive aphasia and motor-neuron disease-related FTD.

Overall, current data on cardiovascular autonomic failure in tauopathy-related cognitive impairment are scarce, provide contrasting results, and remain limited by a substantial heterogeneity [68]. The frequency of overt neurogenic OH in people with clinically ascertained tauopathies remained low in the reviewed studies and the role of age-related confounders, such as cardiovascular comorbidities, medications, mixed pathology, or aging itself, remains to be clarified [67, 68, 70].

While the exact neuropathological substrate of cardiovascular autonomic dysfunction in the tauopathies has not yet been unravelled, both serological and imaging studies indicate intact post-ganglionic sympathetic fibers in these diseases [55–57, 64]. Normal supine and standing plasma NE levels were reported in individuals with AD, as well as in all but one with bvFTD [64], while ^123^I-MIBG-SPECT studies showed intact cardiac sympathetic innervation in people with AD [55–57]. Cardiac ^123^I-MIBG-SPECT has been therefore proposed as useful tool to differentiate both bvFTD and AD from Lewy body disorders related dementia [55–57]. Ultimately, the presence of otherwise unexplained neurogenic OH with evidence of post-ganglionic sympathetic denervation at ^123^I-MIBG-SPECT in a cognitively declined individual points towards an α-synucleinopathy, most likely DLB [55, 67, 68, 71].

Although the current consensus criteria consider dementia as a non-supportive feature pointing against a diagnosis of MSA [4], there is growing evidence that cognitive impairment in fact represents an important non-motor feature affecting about one-third of people with MSA [27, 41, 72]. Recent neuropathological series found that up to one in seven individuals who received an ante-mortem MSA diagnosis in fact turned out to suffer from DLB at post-mortem examination [41, 49]. Early and severe autonomic failure and an “atypical” presentation with only mild cognitive impairment were found to represent the main factors determining an ante-mortem misdiagnosis of MSA in DLB individuals [49]. Beyond DLB-specific clinical red flags, such as early, rapidly progressive cognitive decline with marked fluctuations in attention and alertness, ancillary findings indicating a peripheral pattern of ANS lesion, i.e. cardiac denervation at ^123^I-MIBG-SPECT, may help the differential diagnosis of DLB from MSA, even in cases with a rather “atypical” presentation [14, 19, 34, 41, 49, 56, 57].

By contrast, PD dementia and DLB present largely overlapping features of cardiovascular autonomic failure, despite this is considered more prevalent and severe in the latter [20, 55]. Ultimately, the arbitrary cut-off of one year (“1-year rule”) remains useful in discriminating DLB from PD with dementia in clinical practice [19].

### Cerebellar ataxia

Ataxia, originally from ancient Greek meaning “absence of order”, is defined by an impaired coordination of voluntary movements, which may result from sensory, vestibular or cerebellar impairment, or a combination thereof [73]. Individuals may present with acquired ataxia due to a wide range of symptomatic causes, which should be considered in the diagnostic work-up and sought for with a careful history taking, neurological examination, imaging, and laboratory tests [74]. In absence of apparent exogenous or endogenous non-genetic causes, ataxia may be hereditary or idiopathic [74].

The group of idiopathic late-onset cerebellar ataxias (ILOCA) includes MSA-C as well as sporadic adult-onset ataxia of unknown aetiology (SAOA) [74]. According to the current definition, SAOA is a clinical diagnosis of exclusion, which is given if acquired and genetic causes (either by exclusion of a causative genetic mutation or a presumably negative family history, if genetic testing is not available) are ruled out and the diagnostic criteria for MSA [4] are not met [74]. Absence of severe autonomic failure over the disease course eventually represents the discriminating element between SAOA and MSA [74, 75]. Studies however showed that a substantial proportion of people initially labelled as SAOA may develop overt autonomic failure during the disease course, i.e. phenoconvert to MSA [75–77]. In contrast to previous reports that milder urogenital symptoms, i.e. urinary urgency, frequency, or sexual dysfunction, already discriminate MSA from SAOA at initial presentation [75], a high prevalence of these was recently found at baseline examination of individuals with SAOA who did not convert to MSA during follow-up [76]. As early as 2007, it was shown that mild cardiac parasympathetic abnormalities may be observed in up to 58% of SAOA individuals at CAFTs [78], but no relevant orthostatic BP falls fulfilling the criteria for neurogenic OH [8]. Milder falls in BP upon standing, as well as pathological cardio-vagal responses to the Valsalva manoeuver and deep breathing, may in fact indicate future phenoconversion to MSA, whereas normal CAFT results at initial assessment may direct to genetic testing, especially if a SAOA diagnosis was based on a negative family history [74, 76]. Cardiovascular autonomic failure can therefore be considered the most specific sign guiding the differential diagnosis of ILOCAs towards MSA [76]. However, because its sensitivity increases with the disease duration, the absence of overt cardiovascular autonomic failure at early stages does not exclude a phenoconversion to MSA later on [74–76].

Both male and, less frequently, female individuals with 55–200 CGG repeat expansions of the FMR1 gene may develop the fragile X-associated tremor ataxia syndrome (FXTAS), which clinically manifests with progressive cerebellar ataxia, prominent tremor, as well as cognitive impairment, neuropathy, l-Dopa-unresponsive parkinsonism and autonomic failure [74, 79–81]. The latter may include urogenital and gastrointestinal dysfunction, as well as cardiovascular autonomic features, e.g. post-prandial hypotension and symptomatic neurogenic OH [79–81]. Due to the overlap in symptoms, the European MSA study group screened for FMR1 repeat expansions in a large cohort of clinically diagnosed MSA individuals and found them in ≤ 1% of cases (*n* = 4/426), but in 4% of those presenting with a MSA-C phenotype (*n* = 3/76) [80]. While urogenital dysfunction was found in all misdiagnosed cases, neurogenic OH was present only in one [80]. To date, evidence of cardiovascular autonomic failure in FXTAS is limited to few case reports [79–81] and despite occurring less frequently than urogenital dysfunction, it may still lead to a MSA misdiagnosis, especially if cerebellar features predominate [80]. An unusually slow progression, predominant intention tremor or peripheral neuropathy, as well as a familial clustering of mental retardation, may eventually prompt genetic testing for FMR1 repeat expansions in people with ILOCA [80].

GAA triplet expansions in the FXN gene cause the single most common hereditary cause of ataxia in Caucasian natives, that is Friedreich’s ataxia (FRDA) [82]. Recent evidence from a single-center, but well-characterized cohort of genetically confirmed FRDA individuals and controls without diabetes mellitus or symptomatic cardiomyopathy, showed a high prevalence of urinary symptoms [82], but unremarkable CAFT results, indicating that cardiovascular autonomic failure is not typical of the FRDA clinical spectrum [82, 83]. However, because diabetes mellitus and hypertrophic cardiomyopathy may frequently occur in people with FRDA, diabetic autonomic neuropathy or non-neurogenic forms of OH may develop in some individuals, especially in case of poor glycaemic control, overt heart failure or anti-hypertensive treatments [82].

The cerebellar ataxia, neuropathy, vestibular areflexia syndrome (CANVAS) recently emerged as an additional cause of late-onset ataxia [84]. Cortese et al. identified biallelic intronic AAGGG expansions in the RFC1 gene as CANVAS’ underlying cause [85]. The phenotypical characterization of a large cohort of people with genetically confirmed CANVAS showed that autonomic dysfunction can be part of the CANVAS clinical spectrum, with up to 13% of individuals reporting symptoms suggestive of OH [85, 86]. At autonomic function testing, every fourth individual of the abovementioned CANVAS cohort had a pathological regulation of cardio-vagal reflexes and every third showed sympathetic abnormalities, either during skin sympathetic reflex, handgrip testing or upon orthostatic challenge, but neither the hemodynamic indices, applied reference values nor the prevalence of OH were reported [86]. In contrast to MSA, however, autonomic dysfunction in CANVAS individuals was generally considered mild, developed late in the disease course, and rarely resulted in significant disability, i.e. overt neurogenic OH [84, 86]. Biallelic AAGGG expansions were also not identified in clinically diagnosed MSA individuals [4], suggesting that the slower disease progression and additional signs, such as prominent vestibular dysfunction and sensory neuropathy, prevent people with CANVAS from being misdiagnosed with MSA [84, 86, 87].

The group of autosomal dominant spinocerebellar ataxias (SCA) includes various subtypes to be considered in the differential diagnosis of late-onset cerebellar ataxia, even in the case of negative family history [74, 88]. Because autonomic dysfunction as well as parkinsonism may occur in some SCA subtypes (e.g. SCA 1, 2, 3, 6, and 17), difficulties may arise in the differential diagnosis of both MSA-C and MSA-P [88]. Only recently, it was shown that 7.3% (*n* = 22/302) of individuals with possible and probable MSA [4] in fact carried mutations for dentatorubropallidoluysian atrophy, SCA 1, 2, 3, 6, and 17, with the latter accounting for 60% of the genetic mimicries (*n* = 13/22) [89]. Urinary incontinence, or at least frequency, were present in all misdiagnosed cases, whereas almost half (*n* = 10/22; 80% of those with SCA 17 mutations) showed severe BP falls of ≥ 30/15 mmHg or reported orthostatic dizziness [89]. Two other studies found that overt OH did not occur in a SCA 2 only—as well as a mixed cohort including individuals with SCA 1, 2, 3, 6 and FRDA mutations, with however pathological cardio-vagal responses to the Valsalva manoeuver and deep breathing in more than the half of cases [76, 90]. Interestingly, earlier reports indicated that cardiovascular autonomic failure with OH may be found in up to 25% people with SCA 3 mutations, with one study also reporting cardiac sympathetic denervation at ^123^I-MIBG-SPECT [91, 92]. At present, neurogenic OH seems to yield a limited diagnostic accuracy for distinguishing MSA-C from autosomal dominant cerebellar ataxia, particularly SCA 3 and 17, but further studies in larger independent cohorts with combined CAFT and cardiac ^123^I-MIBG-SPECT are needed to clarify this issue [34, 76, 89–92]. Whereas it is not generally recommended to perform genetic testing in individuals with clinically established MSA [4], this may be considered in case of positive family history, isolated cerebellar atrophy at neuroimaging or mild, non-progressive forms of autonomic failure [88, 89].

Neither supine-/nocturnal hypertension nor post-prandial- or exercise-induced hypotension currently contribute to the differential diagnostic work-up of cerebellar ataxia.

### Summary

Even after five years of disease duration, a significant proportion of individuals initially presenting with autonomic failure will eventually phenoconvert to a manifest CNS α-synucleinopathy, raising the question whether PAF represents an entity of its own or rather a prodromal stage of MSA, PD, or DLB [2, 29–31]. In this context, “isolated” would be a more cautious term to use instead of “pure” autonomic failure, leaving the possibility open for a future clinical phenoconversion. The predominant site of autonomic lesion, i.e. central versus peripheral, together with other clinical and wet biomarkers was shown to reliably predict phenoconversion from isolated autonomic failure to MSA or a Lewy body disorder, either PD or DLB [2, 29–31]. Early identification and differentiation of these individuals are of high scientific and clinical interest, since it may enable their enrolment into disease-modifying clinical trials at a stage in which the neurodegenerative process is already present, but has not caused any motor disability yet [29, 30]. Future studies with large sample sizes and longer follow-up time are needed to confirm the existing-, but also integrate additional imaging and wet predictors of phenoconversion. On the other hand, it is intriguing to speculate that individuals retaining a stable PAF phenotype may have genetic traits protecting them from developing CNS α-synuclein aggregates, equally deserving further investigations to understand the mechanisms underlying α-synuclein-related neurodegenerative processes [2].

In individuals presenting with parkinsonism, the presence of overt cardiovascular autonomic failure allows to exclude both PSP and CBD with high specificity [34, 39–41], while the pattern of ANS lesion distribution in conjunction with the time course of autonomic onset may guide the differential diagnosis between MSA and Lewy body disorders [3, 11, 14, 34, 41, 52–57].

Compared to parkinsonism, there is only limited data available in cognitively impaired individuals, especially with regard to tauopathy-related dementia [34]. While it remains to be clarified whether τ-related neuropathological changes involving central autonomic relays may cause overt cardiovascular autonomic failure [67, 68, 70], an otherwise unexplained neurogenic OH with evidence of cardiac sympathetic denervation yields a high negative predictive value for tauopathy-related dementia and rather points to an α-synucleinopathy-related dementia [55, 67, 68, 71]. PD with dementia and DLB are still separated by the one-year rule [19].

In individuals with ILOCA, presence of cardiovascular autonomic failure with OH allows to distinguish MSA-C from SAOA [34, 74]. By contrast, with exception of FRDA and SCA 2, cardiovascular autonomic failure may have a limited contribution to the differential diagnosis of MSA versus hereditary cerebellar ataxias, particularly SCA 3 and 17 [34, 76, 80, 84, 89]. Mild forms of autonomic failure have been reported in CANVAS and FXTAS, which however represent unlikely MSA misdiagnoses, because of additional disease-specific characteristics and a generally slower disease progression [80, 84, 86]. An atypical MSA presentation, with mild or non-progressive forms of autonomic failure, may eventually raise the suspicion of a genetic lookalike and prompt additional testing, even in individuals with negative family history [76, 80, 88, 89].

Despite representing important clinical findings with major therapeutic implications [93], supine and nocturnal hypertension, as well as post-prandial- and exercise-induced hypotension, currently do not aid the differential diagnosis of autonomic disorders. The spectral analysis of HR variability in the time and frequency domain represents another measure of cardiovascular autonomic function [94–96]. Due to considerable overlaps among neurodegenerative disorders, spectral HR analysis does not provide reliable implications for their differential diagnostic work-up either [94, 95, 97]. However, a reduced HR variability in the absence of overt neurogenic OH may indicate incipient baroreflex dysfunction and eventually make physicians alert of an increased susceptibility to BP active medications in affected individuals.

The major shortcomings of the reviewed literature were the lack of validation in neuropathologically or genetically confirmed series. Significant heterogeneity in methodology, i.e. discrepancies in the definition of neurogenic OH or the performance of CAFT, but also the generally scant or very early biomarker data (e.g. plasma NE measurements; ^123^I-MIBG-SPECT) further limit the generalizability of the reported findings, identifying the areas of future research.

In clinical practice, CAFT should be carried out following the international consensus recommendations [13, 97, 98]. The prevalence of OH, comorbidities, and polypharmacy typically increases with age [9, 10, 99, 100]. When screening elderly subjects, attention must be paid to the fact that non-neurogenic factors, such as concomitant heart failure, other cardiac diseases, medication or other frequent causes of neurogenic OH, e.g. diabetic neuropathy, may influence the global cardiovascular performance [9, 10, 99, 100]. Advanced motor- and cognitive impairment may also negatively impact on the execution of CAFTs, especially those examinations requiring an individual’s active cooperation, such as the Valsalva manoeuver or deep breathing [13, 65, 76, 91]. This may lead to false abnormal CAFT results. Moreover, cognitive impairment may interfere with the perception and the communication of symptoms of orthostatic intolerance, with a substantial risk of eventually under-recognizing the presence of cardiovascular autonomic failure or underestimating its impact on the quality of life of demented people [64–66].

In conclusion, the presence of cardiovascular autonomic failure significantly contributes to the diagnostic work-up of the α-synucleinopathies and related disorders. Given its rapidly progressive, fatal disease course, MSA emerges as the most crucial differential diagnosis to recognize or exclude. Beyond the differential diagnostic implications arising from the presence of cardiovascular autonomic failure, the establishment of an appropriate therapeutic regimen is of utmost importance in order to reduce the symptomatic burden and avoid short and long-term complications of untreated BP dysregulation [93]. Screening for neurogenic OH at bedside with simple supine to standing HR and BP measurements [101, 102] and confirmation of the diagnosis with CAFT should therefore always be considered if cardiovascular autonomic failure is suspected.

## Data Availability

Data sharing is not applicable to this article as no datasets were generated or analyzed during the current study.
